# Oxidative eustress: On constant alert for redox homeostasis

**DOI:** 10.1016/j.redox.2021.101867

**Published:** 2021-01-20

**Authors:** Helmut Sies

**Affiliations:** a Institute of Biochemistry and Molecular Biology I, Faculty of Medicine, Heinrich-Heine-University Düsseldorf, Düsseldorf, Germany; b Leibniz Research Institute for Environmental Medicine, Düsseldorf, Germany

**Keywords:** Redox biology, Steady-state, Homeodynamics, Redox landscape, Hydrogen peroxide, Oxidative stress

## Abstract

In the open metabolic system, redox-related signaling requires continuous monitoring and fine-tuning of the steady-state redox set point. The ongoing oxidative metabolism is a persistent challenge, denoted as oxidative eustress, which operates within a physiological range that has been called the ‘Homeodynamic Space’, the ‘Goldilocks Zone’ or the ‘Golden Mean’. Spatiotemporal control of redox signaling is achieved by compartmentalized generation and removal of oxidants. The cellular landscape of H_2_O_2_, the major redox signaling molecule, is characterized by orders-of-magnitude concentration differences between organelles. This concentration pattern is mirrored by the pattern of oxidatively modified proteins, exemplified by S-glutathionylated proteins. The review presents the conceptual background for short-term (non-transcriptional) and longer-term (transcriptional/translational) homeostatic mechanisms of stress and stress responses. The redox set point is a variable moving target value, modulated by circadian rhythm and by external influence, summarily denoted as exposome, which includes nutrition and lifestyle factors. Emerging fields of cell-specific and tissue-specific redox regulation in physiological settings are briefly presented, including new insight into the role of oxidative eustress in embryonal development and lifespan, skeletal muscle and exercise, sleep-wake rhythm, and the function of the nervous system with aspects leading to psychobiology.

## 1 Introduction

Maintenance of redox homeostasis is a continuously ongoing challenge. Constant surveillance is a hallmark for establishment and maintenance of redox homeostasis, which is more precisely called ‘homeodynamics’ because of its underlying dynamic nature [1]. Recent research progress in redox biology revealed a redox architecture of physiological function [2], which is organized according to a set of principles denoted as the ‘Redox Code’ [3]. One of these principles is that of activation/deactivation cycles of redox metabolism, especially involving H_2_O_2_, which with other molecular signaling agents supports spatiotemporal sequencing in differentiation and life cycles of cells and organs [3]. The wider field of redox signaling has been reviewed extensively (see, for example, Refs. [[4], [5], [6], [7], [8]]).

Cellular redox dynamics is intimately linked to sophisticated structural events at the molecular and cellular level. The latter is illustrated by the continual reshaping, at a seconds-timescale, of the cristae at the mitochondrial inner membrane, revealed by super-resolution nanoscopy [9]. Such reshaping, in turn, is linked to changes in mitochondrial supercomplex formation (see Ref. [10]). The underlying molecular monitoring events include redox parameters, and these precede the structural changes at timescales considerably shorter than the seconds range. H_2_O_2_ contributes to stability of the mitochondrial redox network [11], and there is an integrated redox network at the level of the cell and its organelles for monitoring homeostasis [12,13]. Maintenance is achieved by constant monitoring redox activity in ‘oxidative eustress’ [14].

Here, we start with the conceptual background on redox homeostasis, characterizing the metabolic steady-state. This is followed by a generalized snapshot of the cellular redox landscape with focus on H_2_O_2_, the central redox signaling metabolite [[15], [16], [17], [18]]. Short-term (non-transcriptional) and longer-term (transcriptional/translational) mechanisms will be outlined. Examples of emerging redox research areas will be presented, focusing on diurnal (circadian) rhythm, sleep-wake cycles, embryonal development and lifespan, skeletal muscle and exercise, and some aspects of the nervous system which touch on molecular relationships in psychobiology.

## 2 Conceptual: open system and maintenance of steady-state

Von Bertalanffy [19] pioneered the biophysics of flow-equilibrium, which is called ‘steady-state’: “*Living systems are open systems, maintaining themselves in exchange of materials with environment, and in continuous building up and breaking down of their components”* [19]. The open metabolic system requires continuous monitoring of inflow and outflow to minimize deviation from the steady-state set point. In redox regulation, this refers to physiological oxidative stress, or oxidative eustress (see Ref. [20]). The physiological range of excursions from the set point has been called the ‘Homeodynamic Space’ [21], the ‘Goldilocks Zone’ [22] and the ‘Golden Mean’ [23].

Furthermore, the ever-changing metabolic conditions require appropriate adjustment of the steady-state set point, *i.e*. the target value of reduction-oxidation in the spatiotemporal context. This is epitomized by Selye's adaptive stress concept [24]. Prigogine [25] analyzed time structure and fluctuations, which he called ‘dissipative structures’ in non-equilibrium thermodynamics. In biology, the idea of Claude Bernard's ‘milieu intérieur’ [26] has found attraction in the terms ‘resilience’ and ‘allostasis’. Resilience denotes the ability to return to the original condition, to bounce back, whereas allostasis refers to the achievement of stability through change to a new set point [27], which is a *contradictio in adjecto*. A related term is ‘adaptive homeostasis’, formulated by Davies [28].

Thus, mechanisms for maintenance of homeostasis can be divided into reactive (feedback, counterregulation) and predictive (feedforward, anticipatory) modes.

**Fig. 1:**
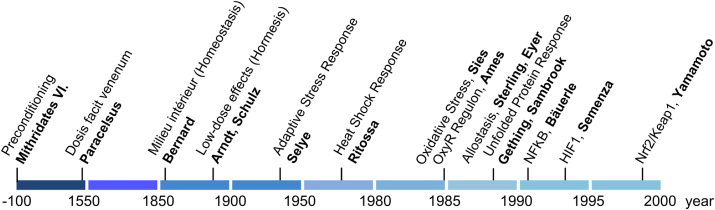
Timeline of concepts of stress and adaptive stress responses. Mithridates VI [132] and Paracelsus [133] had early insight that the dose matters in deciding beneficial versus harmful outcome. Bernard's concept of the ‘milieu intérieur’ [26] received the name ‘homeostasis’ [134], and the Arndt-Schulz [135] rule received the name ‘hormesis’ [136]. The 20^th^ century brought the adaptive stress syndrome [24], heat shock response [137], oxidative stress [138], OxyR [139], allostasis [27], unfolded protein response [140], and the major mammalian master regulators NF-kB [141], HIF1 [142], and Nrf2/Keap1 [143]. From Ref. [20].

As for the latter, the capability of preconditioning in response to endogenous and exogenous (‘exposome’) cues, summarized under the terms ‘hormesis’ [29] and more specified ‘mitohormesis’ [30], is important for adaptive stress responses. Fig. 1 gives a timeline of the concepts of stress and stress responses.

Selye introduced the distinction between eustress and distress in 1975 [31], and oxidative eustress in molecular terms has become of interest in recent years to signify physiological, not harmful, oxidative stress [17], [20], [32a], [32], [33], [34], [35], [36].

## 3 Spatiotemporal controls

Cellular organization is characterized by subcellular compartmentation and gradients. Interestingly, the fast-acting non-transcriptional signaling agents occur at about nM concentration; H_2_O_2_, Ca^2+^ and pH are shown in Table 1. (It may be pointed out that pH = 7 signifies [H^+^] = 100 nM H^+^.) Other important signaling entities such as ^**.**^NO [37] and H_2_S [38] also occur at nM physiological concentration, constituting a ‘reactive species interactome’ [39,40]. Furthermore, there is redox signaling by reactive electrophiles for maintenance of the nucleophilic tone [23,41].

**Table 1:** **Fast-acting signaling agents H**_**2**_**O**_**2**_**and Ca**^**2+**^**occur at nanomolar concentration.** Proton concentration [H^+^] is at similar concentration. Columns are in nanomolar [nanomol/L] and as p = –log_10_ [mol/L], to illustrate the similar physiological ranges for H_2_O_2_, Ca^2+^ and H^+^. Numbers refer to generic resting cells, ranges not shown; exact quantification in subcellular organelles is yet to be obtained.

	H_2_O_2_		Ca^2+^		H^+^	
	nM	(pH_2_O_2_)	nM	(pCa)	nM	(pH)
‘Overall cellular’^a^	10	(8) [128,129]	100	(7)	100	(7)
Cytosol	0.1	(10) [53]	<100	(>7)	100	(7.0)
Mitochondrial matrix	4	(8.4) [54]	<100	(>7)	40	(7.4)
Endoplasmic reticulum	700	(6.2) [52]	500,000	(3.3)	60	(7.2)
Golgi	300	(6.5) [130]	300,000	(3.5)	400	(6.4)
Peroxisome	?		<100	(>7)	7	(8.2) [69]
Lysosome	?		300,000	(3.5) [131]	3200	(5.5)

a For rough orientation only: considerable subcellular variation; Ca^2+^ in various spaces was arbitrarily set to <100 nM.

‘Buffering’ of [Ca^2+^] relies on Ca^2+^ stores in the endoplasmic reticulum and the mitochondrial matrix (see Ref. [42]), and buffering of [H^+^] relies on the action of carbonic anhydrases and respiration. In contrast, there is no ‘buffering store’ of H_2_O_2_ within the cell. Here, control of [H_2_O_2_] relies on swift fine-tuning of enzymatic synthesis and degradation of H_2_O_2_ as well as on gradient control. A role of mitochondria as ‘ROS stabilizing device’ has been postulated [43], and this extends also to control by extramitochondrial sources such as NADPH oxidases [44].

H_2_O_2_ and Ca^2+^ are reciprocally interconnected in numerous signaling processes (see Refs. [[45], [46], [47], [48]]). As listed in Table 2, an illustrative example is that of the transient receptor potential (TRP) channels, which act as biosensors for redox environmental stimuli, being activated by H_2_O_2_, ^**.**^NO and electrophiles [49]. These channels facilitate Ca^2+^ influx, triggering cellular responses. The receptor has a highly reactive cysteine (C621 in TRPA1), the modification of which leads to the opening of the gate [50]. Another example is the suppression of store-operated calcium entry upon oxidation of cysteine 313 in stromal interaction molecule 2 (STIM2), which gates calcium channels [51].

**Table 2:** **Selected examples of physiological oxidative stress (eustress) in life processes.** Cysteine positions attributed to the effects are given. These entries refer to the selected examples discussed in text, as representative of many more in the literature.

Process	Effect	Cysteine (position)	Reference
Sensing of irritants	Trigger for response	C621 in TRPA1	[50]
Store-operated Ca^2+^ entry	Suppression	C313 in STIM2	[51]
Shaping optical tectum	Morphogenesis	C175 in Engrailed-2	[97]

### 3.1 Spatial: cellular redox landscape

**Fig. 2:**
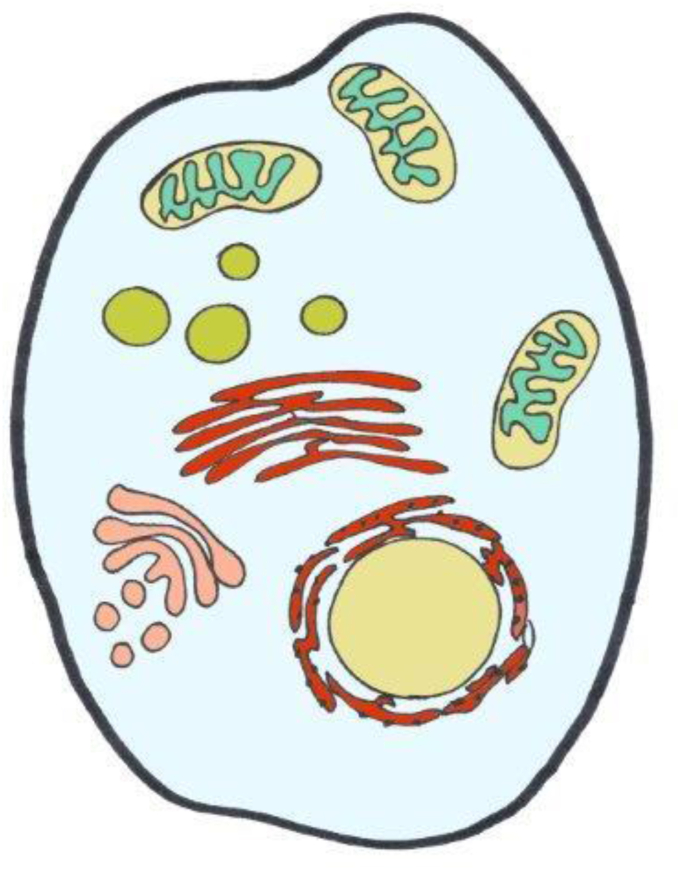
‘Landscape’ of H_2_O_2_ across the cell. Generalized overview of estimated concentrations of H_2_O_2_ in subcellular spaces. Color code is from light blue (80 pM) to blue-green (4 nM), green (ca. 20 nM), brown (300 nM), and red (700 nM) (see Table 1 for References).

The concentration pattern of H_2_O_2_ across cells is depicted in Fig. 2 according to currently available information, which is still limited in terms of calibrated numbers rather than color scales from imaging (see Table 1). The extremes go from the lumen of the endoplasmic reticulum, approaching the μM H_2_O_2_ range [52], down to the cytosol on the lower end, for which as low as 80 pM H_2_O_2_ has been calculated [53]. The mitochondrial matrix is estimated to contain around 4 nM H_2_O_2_ [54]. Little is known about the respective numbers for peroxisomes and lysosomes. Peroxisomal matrix H_2_O_2_ concentration was found to be considerably higher than mitochondrial matrix H_2_O_2_ in experiments with roGFP2-Orp1, but assigning numbers to the peroxisomal data is difficult [55]. Fig. 2 gives a rough orientation; clearly, there are subcellular microdomains [56] or nanodomains [57] with variant steady-state distribution of H_2_O_2_, indicating that the landscape is diversified and subtly readapted upon receiving cues. Thus, a more refined landscape would be considerably more sophisticated due to the intricate inter-organelle relationships known under the name of ‘contactology‘ [[58], [59], [60], [61], [62]].

**Fig. 3:**
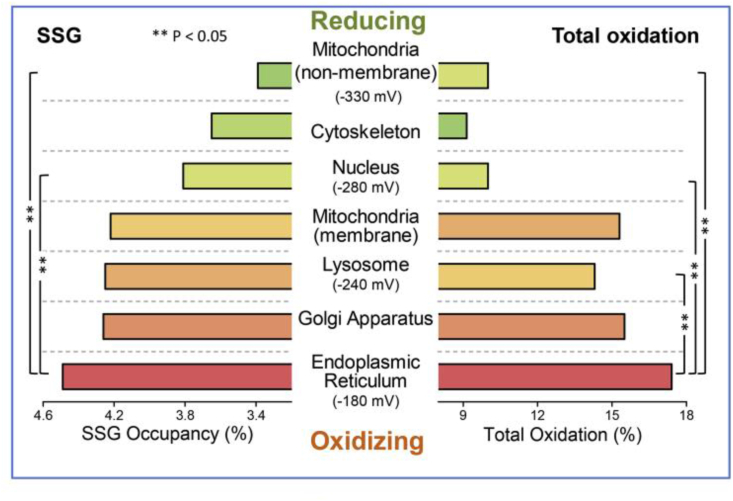
Subcellular distribution of average protein S-glutathionylation (SSG occupancy) and total oxidation. Analysis of the redox proteome of macrophages. From Ref. [63].

It is noteworthy that the ‘landscape’ of H_2_O_2_ resembles the pattern of protein S-glutathionylation: the average occupancy of proteins by S-glutathionylation was recently shown to be highest in the endoplasmic reticulum and lowest in the mitochondrial matrix [63]. The subcellular distribution of S-glutathionylated proteins (Fig. 3) thus resembles that of H_2_O_2_ concentration shown in Fig. 2. It will be of interest to compare this pattern to other types of oxidative posttranslational modifications of proteins (oxPTM), such as the predominantly mitochondrially located Coenzyme A, named S-CoAlation [64], and others like S-persulfhydration, S-acylation, S-nitrosation or S-palmitoylation of redox-sensitive proteins. Functional perspectives of the role of these oxPTMs are being elucidated, *e.g*. as given for S-glutathionylation [65,66] or S-CoAlation [67]. A further level of refinement concerns the reactivity of protein cysteine thiols: the pK_a_ of glutathione persulfide (GSSH) is about 3.5 units lower than that of glutathione (GSH) [68], making GSSH much more reactive than GSH at physiological pH. Likewise, at the more basic pH of 8.2 at the peroxisomal matrix [69], thiolate chemistry will be considerably more prevalent than at neutrality. Thus, peculiarities of reactive species and of location can make for more than 1000-fold differences in reactivity, illustrating relationships between redox chemistry and compartmental pH.

### 3.2 Temporal: the redox set point is a moving target

The temporal response can be divided into the seconds range for immediate redox response, and to longer timeframe response. Monitoring at the short time range, the sources of H_2_O_2_ such as NADPH oxidases, mitochondrial respiratory chain complexes and H_2_O_2_-generating enzymes (see Ref. [8]) are turned on instantly in response to metabolic and physical cues. This is without transcriptional/translational activation of gene expression, allowing for tight control of the steady-state set point by feedback loops. An illustrative example is that of the rapid initiation of wound healing, which involves H_2_O_2_, Ca^2+^ and ATP as transcription-independent damage signals operating instantly upon demand [70].

The longer timeframe redox response is on the hours range and beyond, based on transcriptional/translational activation, permitting feedforward as well as feedback regulation. One major feature here is diurnal rhythm, the circadian response pattern. Transcriptional/translational feedback loops (TTFL) form the backbone of the mammalian circadian clock [71,72]. Peroxiredoxins are conserved markers of circadian rhythm [73], and peroxiredoxins have an emerging role as redox relay hubs [74]. The rhythmic expression of peroxiredoxin-6 is cooperatively controlled by the clock protein Bmal1 and Nrf2[75]. The diurnal oscillations of H_2_O_2_, which exert redox control of the CLOCK protein, are influenced by the adaptor protein p66^Shc^, mediating the changes of the H_2_O_2_ set point over the day [76]. Thus, oscillations of H_2_O_2_ blend into the coupled network of circadian clocks [77], making the H_2_O_2_ set point a dynamic moving target.

The circadian rhythm widely impinges on physiology. The molecular basis of redox influence on sleep-wake patterns is beginning to be unraveled. In *Drosophila*, the K_v_ potassium channel ß-subunit was found to couple mitochondrial oxidative events to sleep [78]. Also in *Drosophila*, a bidirectional relationship between sleep and oxidative stress was observed [79]; an increase in sleep in wild-type flies increased their resistance to oxidative stress, while diminishing oxidative stress in neurons shortened sleep, which led the authors to the slogan: “sleep clears ROS, ROS promote sleep” [79]. Sleep loss leads to ‘ROS’ accumulation, and death from sleep loss has been attributed to oxidative stress in the gut [80]. This brief glance at an emerging research field may suffice here.

### 3.3 Gradient control: role of peroxiporins

The concentration of H_2_O_2_ in blood plasma was estimated to be 1–5 μM [81]. Thus, there is a steep gradient across the plasma membrane from outside towards the H_2_O_2_ concentrations inside cells, which is in the nM range (Table 1). Arguably, one could consider the μM extracellular H_2_O_2_ concentration to functionally serve as an ‘H_2_O_2_ store’, which can be tapped into on demand. Likewise, there are substantial H_2_O_2_ gradients between subcellular organelles and the cytosol. Several aquaporins (AQP3, AQP5, AQP8, AQP9, AQP11) facilitate transmembrane diffusion of H_2_O_2_, for which they are more specifically called ‘peroxiporins’ (see Ref. [82]). Work on AQP8 revealed a gating mechanism involving cysteine persulfidation (RSSH), suggesting H_2_O_2_ gradient control by peroxiporins in a redox-dependent manner [83]. AQP11 is localized in the endoplasmic reticulum membrane. It was found that AQP11 efficiently transports H_2_O_2_ to the cytosol, making it a potential regulator of endoplasmic reticulum-based redox signaling [84]. Diurnal aquaporin expression has been found early on in plants [85]. Whether peroxiporins undergo circadian rhythm in mammalian cells seems not to have been examined in detail, but AQP3 in the epidermis has been found to undergo such rhythm [86].

## 4 Functional: cell- and tissue-specific redox regulation; emerging fields

**Fig. 4:**
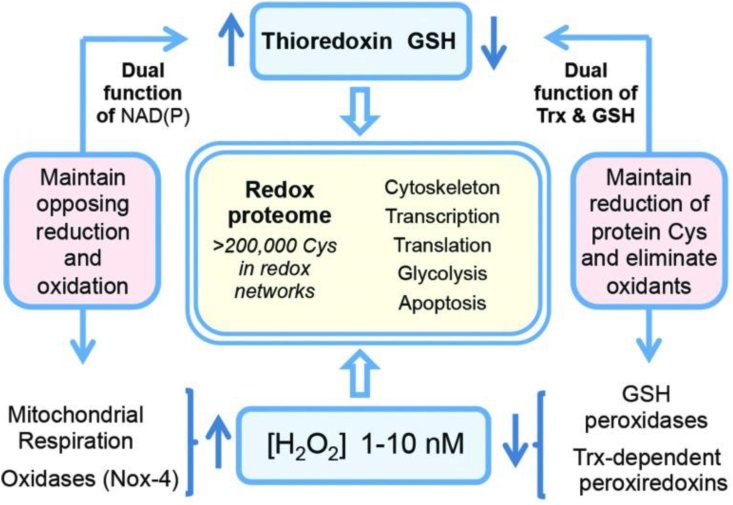
The redox proteome is organized through kinetically controlled thiol switches. From Ref. [3]**.**

Cells and tissues engage in their specialized physiology with redox regulation playing essential roles. In particular, protein-cysteine redox networks within each tissue underlie tissue-specific biology. Fig. 4 recalls a general overview of the redox proteome, which is organized through kinetically controlled thiol switches [3,87,88]. Specificity is conferred by protein-cysteine thiolate reactivity and electrostatic gating [89], [89a] Quantitative mapping of the mouse cysteine proteome *in vivo* using a method called ‘Oximouse’ has opened new perspectives [89]. Assaying this ‘landscape’ revealed that redox regulation of specific proteins is highly tissue-specific. Oximouse redox networks help identifying new pathways of redox regulation (see Fig. 3 in Ref. [89]). Pathways for sensing and responding to H_2_O_2_ at the endoplasmic reticulum have been identified [90].

Selected processes requiring oxidative eustress are now presented in this section, referring to recent literature on these emerging fields, without attempting full coverage of each of these rapidly developing topics. In a recent review [8], other important topics were addressed, including immune system, inflammation and wound repair, the cardiovascular system, insulin sensitivity and pathogenesis of diabetes, aging, and cancer.

### 4.1 Development, lifespan

**Fig. 5:**
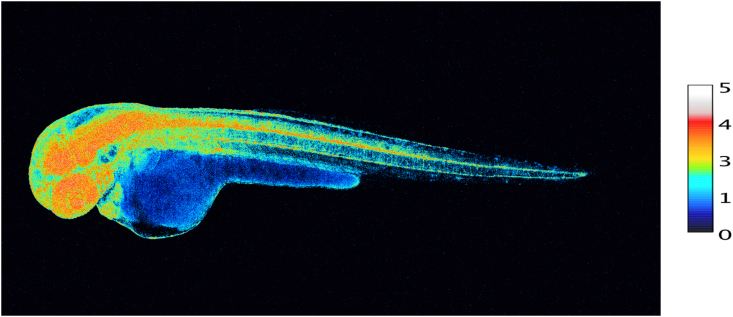
Snapshot of diverse concentrations of H_2_O_2_ in various organs of the intact developing zebrafish embryo at 48 h post fertilization. 100 ng/μL of HyPer7 mRNA was injected in 1-cell stage zebrafish embryos. Scale bar, 100 μm. Embryo H_2_O_2_ imaging was performed essentially as described in Ref. [97]. H_2_O_2_ concentration is correlated to the YFP500/YFP420 excitation ratio of HyPer7**.** Photo taken by M. Thauvin, kindly provided by Prof. Sophie Vriz, Paris.

During embryogenesis and throughout development, redox signaling contributes to cell fate decisions and organogenesis. Development of an embryo entails considerable changes in redox state [91]. H_2_O_2_ is involved in morphogenesis and cell differentiation [[92], [93], [94]], accompanied by changes in glutathione utilization [95]. Fig. 5 provides a snapshot of the pattern of H_2_O_2_ concentrations in the various organs of a zebrafish embryo at 48 h post fertilization, using the genetically encoded probe, Hyper7. At this stage, H_2_O_2_ concentrations are particularly high in the developing nervous system (brain, retina and spinal cord) as well as in the heart. A role of NADPH oxidases in neuronal development has been substantiated [96]. H_2_O_2_ and the homeoprotein Engrailed synergize to shape the optical tectum, contributing to embryonal patterning in zebrafish [97], which extends earlier findings of the role of H_2_O_2_ on axonal growth cone pathfinding [98]. Information on the role of ‘ROS’ in axonal growth has also been obtained in regeneration studies. After nerve injury, NADPH oxidase-2 is released from macrophages into exosomes, which are incorporated into the injured axons *via* endocytosis [99]. The authors propose the signaling pathway of NOX2-PI3K-p-Akt for axonal regeneration [99].

A molecular link between early-life events, ROS-sensitive epigenetic marks, stress resistance and lifespan has been identified [100]. These effects, observed in *C. elegans* and in *HeLa* cells, were attributed to a global ROS-mediated decrease in a particular developmental histone modification: trimethylation of lysine 4 in histone 3 (H3K4me3) was diminished, causing increased stress resistance [100].

Fate and functions of stem cells are tightly linked to redox homeostasis, deciding between quiescence, self-renewal and differentiation (see Refs. [101], [101a]. Furthermore, the role of oxidants in reproduction is being appreciated [102]. The role of oxidants in assisted reproduction needs to be further elucidated, in order to preserve redox signaling while minimizing oxidative damage [103].

### 4.2 Skeletal muscle: exercise as eustress

H_2_O_2_ is a key signal in skeletal muscle physiology and adaptation to exercise [[104], [105], [106], [107]]. Both short-term immediate response to physical activity [108] and longer-term remodeling and adaptation [106] involve redox signaling by H_2_O_2_. 2-Cys peroxiredoxin-2 was found to be rapidly and reversibly oxidized in response to contractile activity, identifying this protein as an effector in muscle redox signaling [109]. Other effector molecules, likely other reactive peroxidases, will come into play in muscle adaptation [106], and the attenuated exercise response in older individuals may be explained by diminished transient oxidation of effectors such as peroxiredoxin-2[110]. Extracellular superoxide dismutase (SOD3) has been implicated in dampening oxidative challenge during exercise [111]. The loss of muscle mass (cachexia) in cancer is related to altered redox homeostasis [112].

### 4.3 Oxidative eustress, the nervous system and psychobiology

The brain utilizes redox signals for many functions, the hypothalamus being the ‘master orchestrator’ [113]. There is redox crosstalk with metabolic signaling at the neuron-astrocyte interface [114], and a tight relationship exists between astrocytes and neurons in patterning selenoproteins, which constitute part of redox control [115]. Synaptic plasticity [116], synaptic pruning [117] and glutamate receptor activation [118] are only a few more of many examples of functional use of oxidants in the nervous system, establishing oxidative eustress as pivotal.

The molecular links between psychobiology and redox biology in stress research are moving into focus of mind-body science [119]. A key observation was the distinction of eustress (‘good stress’) from distress (‘bad stress’) in anticipatory cortisol reactivity, and that moderate stress enhances resilience [120]. Interestingly, in critical incident stress training, even decreased salivary cortisol was observed in periods of self-assessed improved performance, *i.e*. psychologically denoted ‘eustress’ [121].

The relationship between psychological stress and mitochondrial function has found attention [122]. A mitochondrial health index (MHI) sensitive to mood and caregiving stress has been established [123]. MHI integrates human leukocyte-derived information on nuclear and mitochondrial DNA-encoded parameters, which reflects the respiratory chain capacity per unit of mitochondrial content. It consists of succinate dehydrogenase/citrate synthase and cytochome *c* oxidase/mitochondrial DNA copy number, respectively. It was found that MHI was correlated to mood parameters [123,124], which were assessed according to protocols for psychological stress and symptoms for depression and anxiety. Mitochondrial dysfunction is closely related to the manifestation of depression [125], and oxidative stress was found to be involved in the linking of psychosocial stress (social isolation, loneliness, effort-reward-imbalance) to cardiovascular disease [126,127].

## 5 Concluding remarks

The main direction of electron flow in aerobic living systems is catabolic, oxidative. However, anabolic metabolism also occurs, reductive: synthetic pathways are driven by reducing equivalents obtained from redox reactions (see Ref. [3]). Relevant for the present context, the main direction of electron flow is associated with a persistent oxidative challenge, characterized as oxidative eustress. The converse departure from the redox set point is towards reduction. Consequently, such process would be called ‘reductive eustress’, as would occur, for example, at hypoxia or with increased metabolic reducing conditions.

In this sense, the concept of physiological ‘resting state’ would be a misnomer, because at that state there is no ‘rest’; instead, it is continuous testing and correcting towards homeostatic balance, *i.e.* homeodynamics. The ‘resting state’ can be likened to the ‘stand-by’ or ‘idling’ position in an automobile, which is ready to accelerate, rather than to ‘motor off’, the shutdown of the engine. The three now classical major redox-responsive molecular switches epitomize this fact: the NFkB, Nrf2/Keap1, HIF systems operate on constant alert by coupling oxidant and electrophile status to their level of activation.

## Declaration of competing interest

I declare no conflict of interest.
